# The Oropouche fever in Latin America: a hidden threat and a possible cause of microcephaly due to vertical transmission

**DOI:** 10.3389/fpubh.2025.1490252

**Published:** 2025-03-26

**Authors:** Nathália Mariana Santos Sansone, Matheus Negri Boschiero, Luiz Felipe Azevedo Marques, Fernando Augusto Lima Marson

**Affiliations:** ^1^Laboratory of Molecular Biology and Genetics, São Francisco University, Bragança Paulista, SP, Brazil; ^2^Laboratory of Clinical Microbiology and Genetics, São Francisco University, Bragança Paulista, SP, Brazil; ^3^LunGuardian Research Group—Epidemiology of Respiratory and Infectious Diseases, São Francisco University, Bragança Paulista, SP, Brazil; ^4^Medical Resident in Infectious Disease at the Federal University of São Paulo, São Paulo, SP, Brazil

**Keywords:** *Culicoides paraensis*, dengue, diagnosis, epidemiology, pregnancy, viral infection, Zika virus

Dear Editor, Latin America (LATAM) is facing a severe outbreak of arbovirus, particularly dengue fever (1, 2). In addition, the cases of Oropouche fever (OF), a zoonotic disease caused by the Oropouche virus (OROV), are increasing (3). The OROV is a member of the *Bunyaviridae* family. Its viral genome is composed of three segments of negative-sense single-stranded ribonucleic acid (RNA). Previous outbreaks described between 2022 and 2024 were associated with viral rearrangements resulting in a reassortant virus, with genomic segments from different previously circulating strains (4, 5). Furthermore, a genomic rearrangement has been observed between the OROV and the Iquitos virus, both classified under the *Orthobunyavirus oropoucheense* species. This classification also included other species: Madre de Dios virus, Oropouche-like virus, and Perdoes virus (4, 5).

Transmitted mainly by the *Culicoides paraensis* midge, OF can also spread through other arthropods such as *Aedes serratus, Culex quinquefasciatus*, and *Coquillettidia venezuelensis* (6, 7). First identified in Trinidad and Tobago in the 1950s, OF is endemic to the Amazon region and extends to umpteen Central and South American countries (6).

**Figure 1:**
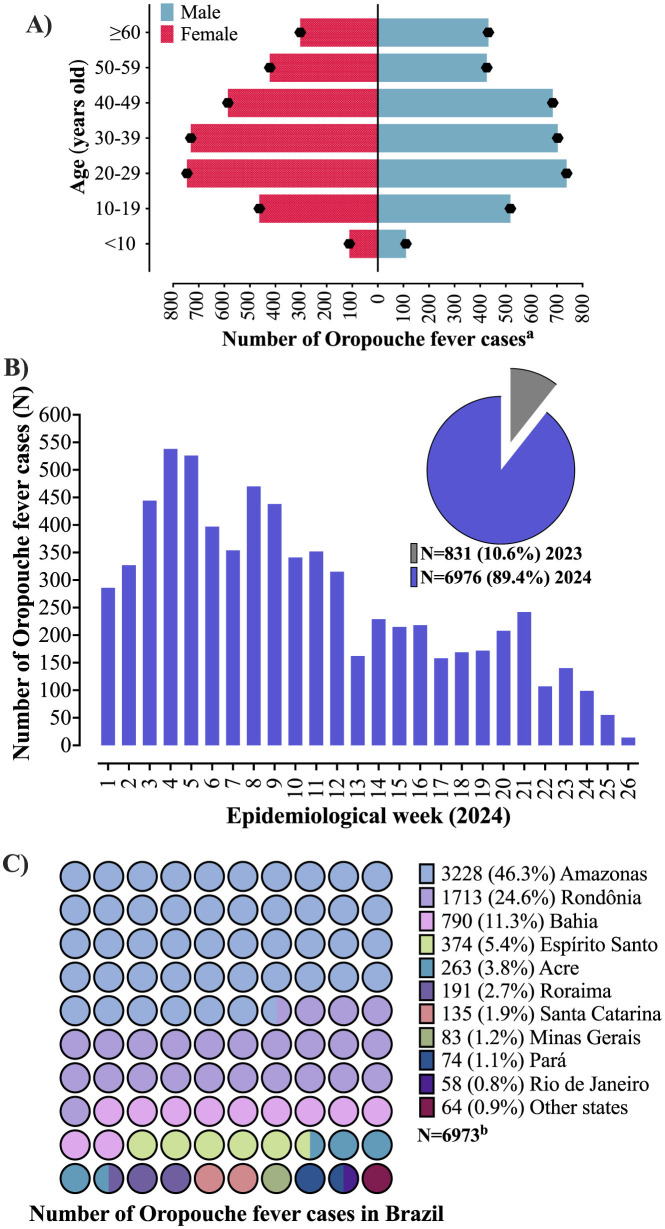
Epidemiology of Oropouche fever in Brazil. **(A)** Frequency of Oropouche fever in Brazil according to gender and age range. **(B)** Number of cases of Oropouche fever in Brazil according to epidemiological weeks. The image also presents the number of cases of Oropouche fever in Brazil registered in 2023. In 2024, data for 26 epidemiological weeks were computed, while in 2023, data for the entire year was collected. In 2023, 457, 178, 152, 43, and 1 cases occurred in the states of Amazonas, Acre, Roraima, Rondônia, and Pará, respectively. **(C)** Brazilian states where cases of Oropouche fever were identified. Other states are presented the following number of cases—Piauí (*N* = 19), Mato Grosso (*N* = 16), Pernambuco (*N* = 9), Amapá (*N* = 7), Ceará (*N* = 5), Paraná (*N* = 3), Maranhão (*N* = 3), Tocantins (*N* = 1), and Mato Grosso do Sul (*N* = 1). Cases from Ceará, Paraná, and Mato Grosso do Sul are still being evaluated to determine the place of infection. ^a^Three individuals did not have information about age or date of birth, ^b^three individuals were probably infected in Bolivia. Data were collected from the website of the Brazilian Ministry of Health, the database of arboviruses. Emergency Operations Center, Weekly Report: Edition No. 21. The data are presented as the number of cases (N) and/or percentage (%).

**Figure 2:**
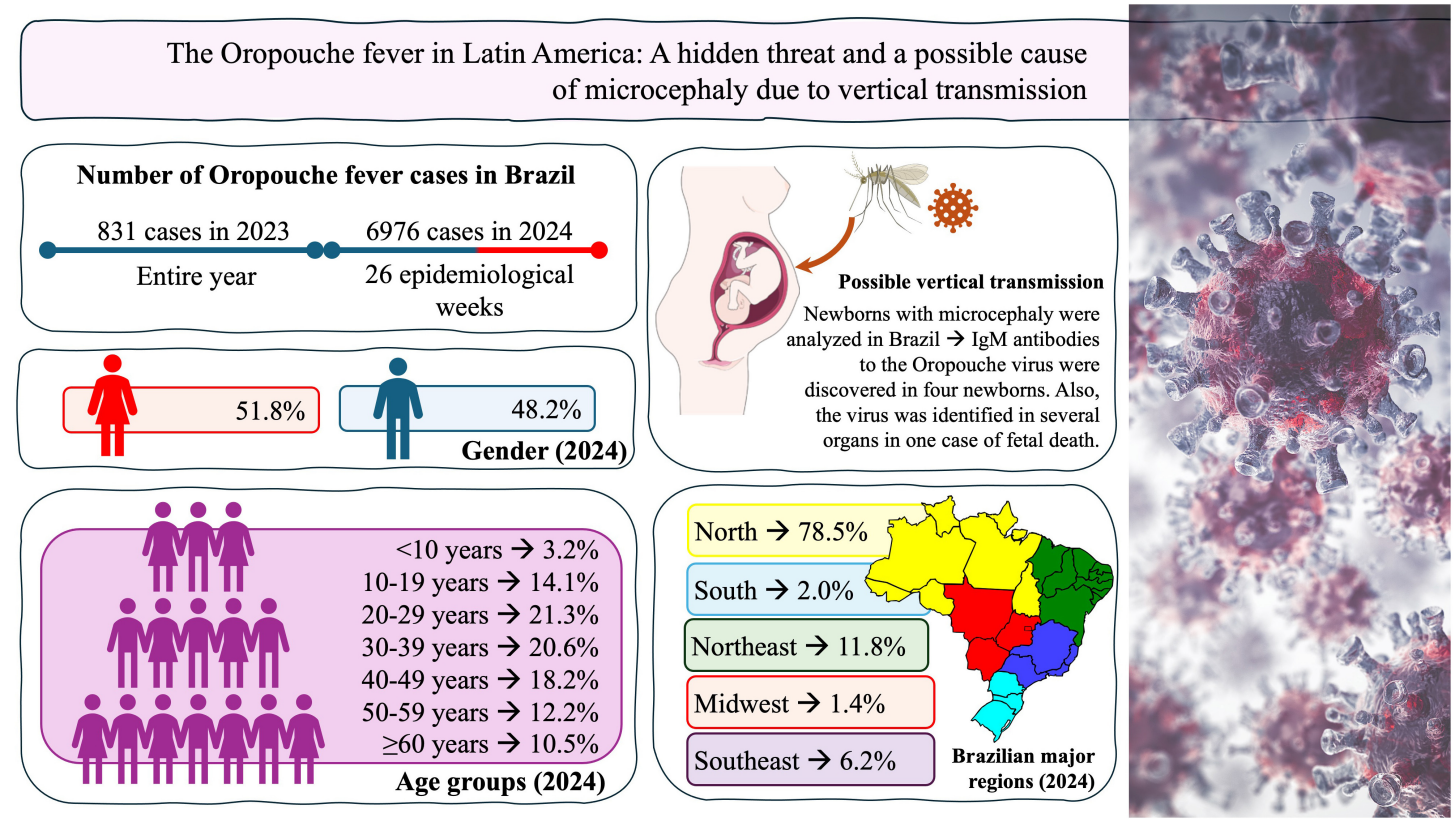
The data is presented as number of cases (N) and percentage (%). Immunoglobulin M (IgM).

As of the 18th epidemiological week in 2024, LATAM reported 5,193 cases of OF, with Brazil accounting for 4,583 cases (88.3%)—according to the Pan American Health Organization. Recent data from the Brazilian Ministry of Health show a dramatic increase to 6,976 cases by the 26th epidemiological week, marking an increase of 839.5% from 831 cases in 2023 (Figures 1A, B; Figure 2). According to a recent Brazilian study, the OROV has been detected in all 26 states of Brazil in 2024. This contrasts with 2023, when the majority of cases were reported in the Amazon and Cerrado regions. Small and medium municipalities from the non-Amazon region were especially affected, with the frequency of cases 3 to 9 times higher than large municipalities (8). Please see the complete data in the Figure 2 and Supplementary Table 1.

OF has also spread to non-endemic regions—extra-Amazonian states—where autochthonous cases have been reported (Figure 1C). Factors such as global warming, deforestation, and flooding may exacerbate mosquito-borne disease outbreaks by modulating the life cycle of mosquitoes and promoting their proliferation (6, 9). Furthermore, it is crucial to screen for viral genomic rearrangements, as these rearrangements in arboviruses have previously been associated with pandemics and human outbreaks.

The symptoms of OF are similar to those of other arboviruses such as dengue and chikungunya, complicating the diagnosis, and routine laboratory tests are scarce (6). The majority of cases are self-limiting, but serious complications such as meningitis and encephalitis can occur (6).

OF can lead to neurological issues such as those described before (7), but only the Zika virus has been definitively linked to microcephaly through vertical transmission (10). On 11 July 2024, the Brazilian Ministry of Health recommended increased surveillance for vertical transmission of the OROV (11). The Evandro Chagas Institute detected immunoglobulin M (IgM) antibodies to the OROV in serum and cerebrospinal fluid samples from four newborns with microcephaly, who tested negative for other arboviruses [Dengue, Chikungunya, Zika, and West Nile virus (12)]. Although the exact link between the OROV and microcephaly is not yet clear, recent findings include a case of fetal death at 30 weeks, with the virus detected in fetal organs, suggesting a potential for vertical transmission (12).

In addition to this discovery, Brazilian researchers traced a recent case series in which they evaluated infants who tested negative for other infectious diseases and congenital malformations, such as microcephaly and arthrogryposis, and their respective mothers for the presence of antibodies against the OROV. A total of 68 samples were collected: 65 from historical cases and 3 from 2024. Of the 68 samples, 6 (8.8%) from newborns exhibited a positive IgM reaction against OROV in the cerebral spinal fluid (6 cases). In addition, 4 cases presented a positive IgM reaction in the serum as well. Of the six newborn samples with a positive IgM reaction to OROV, one case at 44 days of age, had OROV RNA identified through real-time quantitative polymerase chain reaction analysis of cerebral spinal fluid. Furthermore, the patient, who died at 47 days of life, also tested positive for OROV RNA from pleural fluid and tissues from the brain, kidney, and lungs through the real-time quantitative polymerase chain reaction. In this particular case, several modifications of the brain macroscopically and microscopically were described, including necrotic and apoptotic changes in neurons, microglia and astrocytes, vacuolization, and tissue atrophy (13).

The Brazilian Ministry of Health has recommended intensifying the surveillance of pregnancies and newborns in cases of suspected arbovirus infections. This includes monitoring abortions, fetal deaths, and congenital neurological malformations, and collecting relevant biological samples. For protection, pregnant women should avoid areas prone to insects, use fine mesh screens, wear protective clothing, apply repellent, keep their homes clean, and follow local health guidelines if there are confirmed cases in their area (12, 14).

Because it is an emerging arbovirus, in order to deal with the increase in OF cases, it is crucial to control mosquito proliferation and identify mosquito breeding sites (3). LATAM public health agents and governments should collaborate on health policies to promote OF education for better diagnosis and prevention. These efforts could manage local outbreaks, mitigate individual impact, and protect travelers from endemic areas. Continued research into the link between OF and microcephaly in newborns is also essential to understand their relationship. However, in addition to the measures taken to contain arboviruses, there is a need for continuous genomic surveillance for the OROV to monitor possible genomic rearrangements and optimize diagnostic methods. Currently, these methods still depend on complex machinery, especially in developing countries, thus avoiding underreporting. Finally, as occurred with the Zika virus, there is a need for cohort studies, especially prospective ones, with larger samples to assess the long-term complications for affected individuals and babies who may be born with microcephaly or other complications.
