# Supplementary material for: The Oropouche fever in Latin America: a hidden threat and a possible cause of microcephaly due to vertical transmission
Source: Front Public Health. 2025 Mar 26;13:1490252. doi: 10.3389/fpubh.2025.1490252 (PMC11979109; doi:10.3389/fpubh.2025.1490252)
Supplement: Supplementary file 1 [file Table_1.docx]

**Title**: The Oropouche fever in Latin America: A hidden threat and a possible cause of microcephaly due to vertical transmission

**Running title:** Oropouche fever in Latin America

**Supplementary Material — Table**

| **Supplementary Table 1.** Distribution of Oropouche fever cases in Brazil according to gender, age, and Brazilian macroregions. | | | |
| --- | --- | --- | --- |
| **Age groups** | **Male** | **Female** | **Total (%)^a^** |
| <10 years old | 111 | 110 | 221 (3.2%) |
| 10-19 years old | 463 | 518 | 981 (14.1%) |
| 20-29 years old | 746 | 738 | 1484 (21.3%) |
| 30-39 years old | 731 | 703 | 1434 (20.6%) |
| 40-49 years old | 586 | 684 | 1270 (18.2%) |
| 50-59 years old | 422 | 426 | 848 (12.2%) |
| ≥60 years old | 303 | 432 | 735 (10.5%) |
| **Total** | 3362 (48.2%) | 3611 (51.8%) | 6973 |
| **Regions and States** | **2023 (%)** | **2024^b^** | **Total (%)** |
| **North** | 831 (100%) | 5477 (78.5%) | 6308 (80.8%) |
| Acre | 178 | 263 | 441 |
| Amapá | 0 | 7 | 7 |
| Amazonas | 457 | 3228 | 3685 |
| Pará | 1 | 74 | 75 |
| Rondônia | 43 | 1713 | 1756 |
| Roraima | 152 | 191 | 344 |
| Tocantins | 0 | 1 | 1 |
| **Northeast** | 0 | 826 (11.8%) | 826 (10.6%) |
| Bahia | 0 | 790 | 790 |
| Ceará | 0 | 5 | 5 |
| Maranhão | 0 | 3 | 3 |
| Pernambuco | 0 | 9 | 9 |
| Piauí | 0 | 19 | 19 |
| **Midwest** | 0 | 100 (1.4%) | 100 (1.3%) |
| Mato Grosso | 0 | 16 | 16 |
| Mato Grosso do Sul | 0 | 1 | 1 |
| Minas Gerais | 0 | 83 | 83 |
| **Southeast** | 0 | 432 (6.2%) | 432 (5.5%) |
| Espírito Santo | 0 | 374 | 374 |
| Rio de Janeiro | 0 | 58 | 58 |
| **South** | 0 | 138 (2.0%) | 138 (1.8%) |
| Paraná | 0 | 3 | 3 |
| Santa Catarina | 0 | 135 | 135 |

^a^: three individuals did not have information about age or date of birth, ^b^: three individuals were probably infected in Bolivia. Data were collected on the website of the Brazilian Ministry of Health, the database of arboviruses. Emergency Operations Center, Weekly Report: Edition No. 21. The data is presented as the number of cases (N) and/or percentage (%).
